# Physical Activity Attenuates the Genetic Predisposition to Obesity in 20,000 Men and Women from EPIC-Norfolk Prospective Population Study

**DOI:** 10.1371/journal.pmed.1000332

**Published:** 2010-08-31

**Authors:** Shengxu Li, Jing Hua Zhao, Jian'an Luan, Ulf Ekelund, Robert N. Luben, Kay-Tee Khaw, Nicholas J. Wareham, Ruth J. F. Loos

**Affiliations:** 1 MRC Epidemiology Unit, Institute of Metabolic Science, Cambridge, United Kingdom; 2 Department of Public Health and Primary Care, Institute of Public Health, University of Cambridge, Cambridge, United Kingdom; University of Ioannina School of Medicine, Greece

## Abstract

**Background:**

We have previously shown that multiple genetic loci identified by genome-wide association studies (GWAS) increase the susceptibility to obesity in a cumulative manner. It is, however, not known whether and to what extent this genetic susceptibility may be attenuated by a physically active lifestyle. We aimed to assess the influence of a physically active lifestyle on the genetic predisposition to obesity in a large population-based study.

**Methods and Findings:**

We genotyped 12 SNPs in obesity-susceptibility loci in a population-based sample of 20,430 individuals (aged 39–79 y) from the European Prospective Investigation of Cancer (EPIC)-Norfolk cohort with an average follow-up period of 3.6 y. A genetic predisposition score was calculated for each individual by adding the body mass index (BMI)-increasing alleles across the 12 SNPs. Physical activity was assessed using a self-administered questionnaire. Linear and logistic regression models were used to examine main effects of the genetic predisposition score and its interaction with physical activity on BMI/obesity risk and BMI change over time, assuming an additive effect for each additional BMI-increasing allele carried. Each additional BMI-increasing allele was associated with 0.154 (standard error [SE] 0.012) kg/m^2^ (*p* = 6.73×10^−37^) increase in BMI (equivalent to 445 g in body weight for a person 1.70 m tall). This association was significantly (*p*_interaction_ = 0.005) more pronounced in inactive people (0.205 [SE 0.024] kg/m^2^ [*p* = 3.62×10^−18^; 592 g in weight]) than in active people (0.131 [SE 0.014] kg/m^2^ [*p* = 7.97×10^−21^; 379 g in weight]). Similarly, each additional BMI-increasing allele increased the risk of obesity 1.116-fold (95% confidence interval [CI] 1.093–1.139, *p* = 3.37×10^−26^) in the whole population, but significantly (*p*_interaction_ = 0.015) more in inactive individuals (odds ratio [OR] = 1.158 [95% CI 1.118–1.199; *p* = 1.93×10^−16^]) than in active individuals (OR = 1.095 (95% CI 1.068–1.123; *p* = 1.15×10^−12^]). Consistent with the cross-sectional observations, physical activity modified the association between the genetic predisposition score and change in BMI during follow-up (*p*_interaction_ = 0.028).

**Conclusions:**

Our study shows that living a physically active lifestyle is associated with a 40% reduction in the genetic predisposition to common obesity, as estimated by the number of risk alleles carried for any of the 12 recently GWAS-identified loci.

*Please see later in the article for the Editors' Summary*

## Abstract 2

Shengxu Li and colleagues use data from a large prospective observational cohort to examine the extent to which a genetic predisposition toward obesity may be modified by living a physically active lifestyle.

## Editors' Summary

**Background:**

In the past few decades, the global incidence of obesity—defined as a body mass index (BMI, a simple index of weight-for-height that uses the weight in kilograms divided by the square of the height in meters) of 30 and over, has increased so much that this growing public health concern is now commonly referred to as the “obesity epidemic.” Once considered prevalent only in high-income countries, obesity is an increasing health problem in low- and middle-income countries, particularly in urban settings. In 2005, at least 400 million adults world-wide were obese, and the projected figure for 2015 is a substantial increase of 300 million to around 700 million. Childhood obesity is also a growing concern. Contributing factors to the obesity epidemic are a shift in diet to an increased intake of energy-dense foods that are high in fat and sugars and a trend towards decreased physical activity due to increasingly sedentary lifestyles.

However, genetics are also thought to play a critical role as genetically predisposed individuals may be more prone to obesity if they live in an environment that has abundant access to energy-dense food and labor-saving devices.

**Why Was This Study Done?:**

Although recent genetic studies (genome-wide association studies) have identified 12 alleles (a DNA variant that is located at a specific position on a specific chromosome) associated with increased BMI, there has been no convincing evidence of the interaction between genetics and lifestyle. In this study the researchers examined the possibility of such an interaction by assessing whether individuals with a genetic predisposition to increased obesity risk could modify this risk by increasing their daily physical activity.

**What Did the Researchers Do and Find?:**

The researchers used a population-based cohort study of 25,631 people living in Norwich, UK (The EPIC-Norfolk study) and identified individuals who were 39 to 79 years old during a health check between 1993 and 1997. The researchers invited these people to a second health examination. In total, 20,430 individuals had baseline data available, of which 11,936 had BMI data at the second health check. The researchers used genotyping methods and then calculated a genetic predisposition score for each individual and their occupational and leisure-time physical activities were assessed by using a validated self-administered questionnaire. Then, the researchers used modeling techniques to examine the main effects of the genetic predisposition score and its interaction with physical activity on BMI/obesity risk and BMI change over time. The researchers found that each additional BMI-increasing allele was associated with an increase in BMI equivalent to 445 g in body weight for a person 1.70 m tall and that the size of this effect was greater in inactive people than in active people. In individuals who have a physically active lifestyle, this increase was only 379 g/allele, or 36% lower than in physically inactive individuals in whom the increase was 592 g/allele. Furthermore, in the total sample each additional obesity-susceptibility allele increased the odds of obesity by 1.116-fold. However, the increased odds per allele for obesity risk were 40% lower in physically active individuals (1.095 odds/allele) compared to physically inactive individuals (1.158 odds/allele).

**What Do These Findings Mean?:**

The findings of this study indicate that the genetic predisposition to obesity can be reduced by approximately 40% by having a physically active lifestyle. The findings of this study suggest that, while the whole population benefits from increased physical activity levels, individuals who are genetically predisposed to obesity would benefit more than genetically protected individuals. Furthermore, these findings challenge the deterministic view of the genetic predisposition to obesity that is often held by the public, as they show that even the most genetically predisposed individuals will benefit from adopting a healthy lifestyle. The results are limited by participants self-reporting their physical activity levels, which is less accurate than objective measures of physical activity.

**Additional Information:**

Please access these Web sites via the online version of this summary at http://dx.doi.org/10.1371/journal.pmed.1000332.

- This study relies on the results of previous genome-wide association studies The National Human Genome Research Institute provides an easy-to-follow guide to understanding such studies
- The International Association for the Study of Obesity aims to improve global health by promoting the understanding of obesity and weight-related diseases through scientific research and dialogue
- The International Obesity Taskforce is the research-led think tank and advocacy arm of the International Association for the Study of Obesity
- The Global Alliance for the Prevention of Obesity and Related Chronic Disease is a global action program that addresses the issues surrounding the prevention of obesity
- The National Institutes of Health has its own obesity task force, which includes 26 institutes

## Introduction

Changes in our lifestyle, including increased energy intake and lack of physical activity, have been the driving force behind the dramatic increase in obesity prevalence over the past few decades [1]–[3], and increasing physical activity levels have been associated with reduced body fatness and metabolic risk [4]. However, genetic epidemiological studies have firmly established that genetic factors also play a critical role in the development of obesity [5]. Although in theory, genetically predisposed individuals may be more susceptible to obesity in an obesogenic environment, there has been no previous convincing evidence of genotype–lifestyle interactions.

Recent genome-wide association studies (GWAS) have identified 12 loci robustly associated with increased body mass index (BMI) [6]–[10]. We have shown that these loci have a cumulative effect on BMI and on the risk of obesity and that, collectively, these loci can be used to estimate an individual's genetic predisposition to obesity [11]. Although the associations between this set of loci and BMI and risk of obesity were convincing, the variance in BMI explained by these variants is still very small (less than 1%) [11], despite previous observations that BMI has an estimated heritability of 40%–70% [5]. Gene–lifestyle interactions may partly account for the unexplained heritability of BMI [12].

In the current study, we examined whether the genetic predisposition to increased BMI and obesity risk as assessed by a genetic predisposition score, based on the 12 susceptibility loci that were recently identified through GWAS, was modified by self-reported daily physical activity in a large population-based sample from the European Prospective Investigation of Cancer (EPIC)-Norfolk study.

## Methods

### Study Sample

The EPIC-Norfolk study is a population-based cohort study of 25,631 people living in the city of Norwich, UK and its nearby areas. Participants were 39 to 79 y old during the health check between 1993 and 1997. From January 1998, participants were invited for a second health examination, which was attended by 15,786 individuals by October 2000. Full details of the study cohort have been described previously [13],[14]. In brief, trained nurses measured height in centimetres and weight in kilograms and BMI was calculated as weight in kilogram divided by height in meter squared.

DNA of 21,631 individuals, all of white European descent, was available for genotyping. Individuals with prevalent type 2 diabetes (*n* = 522), those with missing values for any of the phenotypes under study (*n* = 617), and those with an absolute annual change of BMI greater than 2 kg/m^2^ or of waist circumference greater than 7 cm (*n* = 62) during a follow-up period of 3–4 y were excluded. In total, 20,430 individuals had baseline data available, of which 11,936 had BMI data at the second health check (Table 1). Those who participated in the second health check-up were leaner (*p* = 1.06×10^−33^) and more physically active (*p* = 3.85×10^−36^). Proportionally more women than men participated in the second health compared to baseline participation (*p* = 0.0004) (Table S1).

**Table 1:** Characteristics of the study samples at baseline and follow-up by sex.

Timing of Measurements	Trait	Men	Women
Baseline	*n*	10,004	10,426
	Age (y)	59.0±9.3	58.5±9.3
	BMI (kg/m^2^)	26.4±3.2	26.1±4.2
	Genetic predisposition score	11.3±2.2	11.2±2.2
	Physical activity level	*n* (%)	*n* (%)
	Inactive	2,989 (29.9%)	3,177 (30.5%)
	Moderately inactive	2,478 (24.8%)	3,349 (32.1%)
	Moderately active	2,333 (23.3%)	2,323 (22.3%)
	Active	2,204 (22.0%)	1,577 (15.1%)
Follow-up	*n*	5,969	5,967
	Age (y)	62.9±9.1	62.1±9.1
	BMI (kg/m^2^)	26.8±3.3	26.3±4.2

Values represent mean ± standard deviation, unless otherwise indicated.

The Norfolk, UK, Local Research Ethics Committee approved the study and all participants gave their informed written consent.

### Physical Activity Assessment

Both occupational (sedentary, standing, physical work, heavy manual work) and leisure-time (cycling, exercise) activities were assessed with a validated self-administered questionnaire [15]. Leisure-time physical activity (hours/week) for both summer and winter was recorded. On the basis of this information, average daily physical activity was calculated as total hours of physical activity per week divided by 7, and this was used to categorise physical activity levels into four groups: inactive (sedentary job, no recreational activity), moderately inactive (sedentary job, <0.5 h/d recreational activity or standing job, no recreational activity), moderately active (sedentary job, 0.5–1.0 h/d recreational activity or standing job, <0.5 h/d recreational activity or physical job, no recreational activity), and active (sedentary job, >1 h/d recreational activity or standing job, >1 h/d recreational activity or physical job with some recreational activity or heavy manual job). This categorization of physical activity levels was predefined and validated against objective measurements of physical activity by means of repeated individually calibrated minute-by-minute heart rate monitoring as described previously [15].

### Genotyping

We genotyped rs3101336, rs10913469, rs6548238, rs7647305, rs10938397, rs925946, rs10838738, rs7132908, rs7498665, rs1121980, rs17782313, and rs368794, representing the obesity susceptibility loci near or in *NEGR1*, *SEC16B*, *TMEM18*, *ETV5*, *GNPDA2*, *BDNF*, *MTCH2*, *FAIM2*, *SH2B1*, *FTO*, *MC4R*, and *KCTD15* genes, respectively. These loci have been identified through recent GWAS for BMI [6]–[10]. Genotype information and genotyping methods for the 12 variants have been reported previously in detail (Table S2) [11]. All variants met the quality control criteria (call rate >95%, blind duplicate concordance >97%, and Hardy-Weinberg equilibrium *p*>0.05).

### Statistical Analyses

Individual SNPs were recoded as 0, 1, and 2 according to the number of BMI-increasing alleles for that particular SNP. The BMI-increasing alleles were defined on the basis of the robust associations of the SNPs with BMI observed in the recent GWAS [6]–[10].

A genetic predisposition score was calculated for each individual by adding up the BMI-increasing alleles of all 12 variants. For individuals with missing genotype data for three or fewer SNPs (97.3% of the total sample), missing genotypes were substituted by the average count of risk alleles for the respective SNP for the purpose of calculating the genetic predisposition score. This resulted in a total number of 19,878 individuals at baseline with a genetic predisposition score of whom 12,201 had full genotyped data for all SNPs and 7,677 individuals had substituted genotypes for 3 or fewer SNPs. Of the 19,878 individuals, 11,651 had data from the second health check. The genetic predisposition score was not different between individuals who did participate in the follow-up and those who did not participate in the follow-up (*p* = 0.606). Sensitivity analyses showed that the results of data with and without substitution of missing genotypes were similar. Here, we only present the results based on the predisposition score with substitution. The genetic predisposition score was normally distributed.

First, we analysed the baseline data cross-sectionally. General linear models (GLMs) were used to test the association of individual SNPs and of the genetic predisposition score with BMI. Logistic regression models were used to examine associations with risk of obesity (18.5≤ BMI <25 kg/m^2^ versus BMI ≥30 kg/m^2^) or overweight (18.5≤ BMI <25 kg/m^2^ versus BMI ≥25 kg/m^2^). Data were adjusted for age, age^2^, sex, and physical activity, and we assumed an additive effect of the BMI-increasing alleles. Interactions between individual SNPs or the genetic predisposition score and physical activity on BMI or risk of obesity or of overweight were examined by including a SNP (or score)-physical activity interaction term in the respective model with the main effects included in the model as well. Analyses were also stratified by physical activity level. We examined the explained variance (*R*-square) of BMI by the genetic predisposition score using GLMs. Furthermore, we examined the predictive value of the genetic predisposition score on obesity risk, stratified by physical activity level by using the area under the receiver operating characteristic (ROC) curve produced by a logistic regression model. We also divided the sample into a “genetically susceptible” group, i.e., those with a genetic predisposition score >11 (median of the genetic predisposition score) and a “genetically nonsusceptible” group, i.e., those with a genetic predisposition score of 11 or less to show interactions between the genetic predisposition and physical activity levels on BMI and obesity risk.

Next, we analysed the data longitudinally with the annual BMI change between the first and second health check as the outcome. GLMs were used to examine the interaction between the genetic predisposition score and physical activity on the annual BMI change, adjusting for age, age^2^, sex, and baseline BMI. All analyses were performed using SAS version 9.1 (SAS Institute Inc.).

## Results

At baseline, each additional BMI-increasing allele in the genetic predisposition score was associated with a 0.154 (standard error [SE] 0.012) kg/m^2^ (*p* = 6.73×10^−37^) increase in BMI, which corresponds to a 445 g increase in body weight for a person 1.70 m tall, but was not associated with physical activity levels (*p* = 0.49). Each increase in physical activity level was associated with a reduction of 0.313 kg/m^2^ (SE 0.025; *p* = 1.2×10^−36^) in baseline BMI, which corresponds to a 904 g decrease in body weight for a person 1.70 m tall.

**Figure 1:**
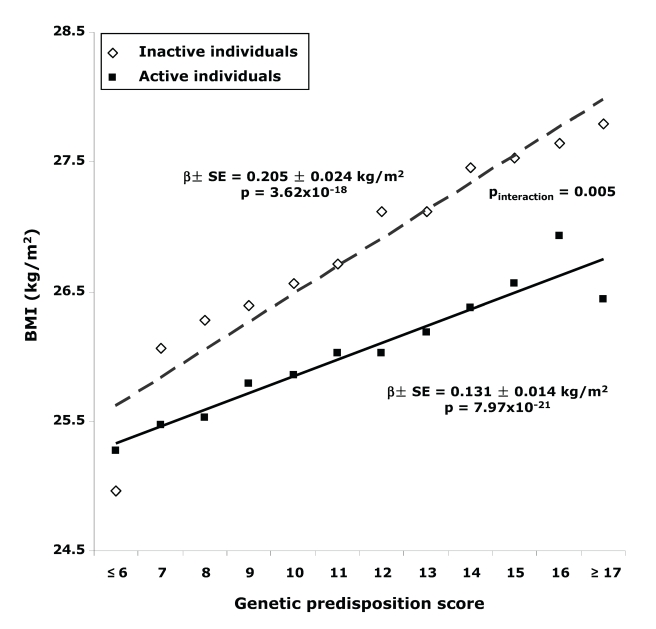
BMI with different genetic predisposition scores in inactive versus active individuals.

Physical activity significantly (*p*_interaction_ = 0.016) modified the effect of the genetic predisposition score on BMI (Table 2). Each additional BMI-increasing allele was associated with an increase of 0.205 (SE 0.024) kg/m^2^ in BMI (*p* = 3.62×10^−18^, equivalent to 592 g in weight) in the inactive group, but the effect was much less in the active individuals (0.126 [SE 0.025] kg/m^2^, *p* = 6.04×10^−7^; 364 g in weight). The effect in moderately active and moderately inactive individuals was intermediate, but more similar to that in the active group. In the combined active group (i.e., the three “active groups” considered together), each additional risk allele increased the BMI with 0.131 (SE 0.014) kg/m^2^ (*p* = 7.97×10^−21^, 379 g in weight), which was significantly less pronounced (*p*_interaction_ = 0.005) than the effect observed in the inactive group (Figure 1). The interaction term remained significant after inverse normal transformation of BMI, suggesting that interaction effects between the genetic predisposition score and physical activity on BMI were not due to unequal variance in different physical activity groups. Similar trends for interaction were observed after further exclusion of individuals with cardiovascular disease (*n* = 1,128) and cancer (*n* = 4,534) (*p*_interaction_ = 0.09 and *p*_interaction_ = 0.05, for using four and two groups of physical activity, respectively).

**Table 2:** Associations of the genetic predisposition score with BMI and risk of obesity in the total population and stratified by physical activity level.

Physical Activity Level	*n*	β^a^ (SE)	*p*-Value	β_weight_ b	*n*_(normal weight)_/*n*_(obese)_	OR^c^ (95% CI)	*p*-Value
Overall	19,878	0.154 (0.012)	6.73×10^−37^	445	7,777/2,798	1.116 (1.093–1.139)	3.37×10^−26^
Inactive	6,004	0.205 (0.024)	3.62×10^−18^	592	2,002/1,100	1.158 (1.118–1.199)	1.93×10^−16^
Moderately inactive	5,667	0.136 (0.022)	1.36×10^−9^	393	2,245/722	1.099 (1.057–1.143)	1.95×10^−6^
Moderately active	4,534	0.130 (0.025)	1.99×10^−7^	376	1,955/558	1.095 (1.047–1.145)	7.10×10^−5^
Active	3,673	0.126 (0.025)	6.04×10^−7^	364	1,575/418	1.092 (1.041–1.147)	3.56×10^−4^

The interaction between the genetic predisposition score and physical activity level was statistically significant for BMI (*p* = 0.016) and risk of obesity (*p* = 0.038).

a Increase in BMI (kg/m^2^) for each additional BMI-increasing allele.

b β, converted to body weight (g) for a person 1.70 tall for each additional BMI-increasing allele.

c Increase in the odds of being obese (BMI≥30 kg/m^2^) versus being normal weight (18.5≤ BMI <25 kg/m^2^) for each additional BMI-increasing allele.

A similar interaction pattern between the genetic predisposition score and physical activity on obesity risk was observed. Each additional BMI-increasing allele was associated with an odds ratio (OR) of 1.116 (95% confidence interval [CI] 1.093–1.139; *p* = 3.37×10^−26^) in the total sample. In the inactive group, each additional BMI-increasing allele was associated with an OR of 1.158 (95% CI 1.118–1.199; *p* = 1.93×10^−16^), which was significantly (*p*_interaction_ = 0.038) greater than the ORs observed for the other physical activity groups (Table 2). In the combined active group, each additional BMI-increasing allele was associated with an OR of 1.095 (95% CI 1.068–1.123; *p* = 1.15×10^−12^) (*p*_interaction_ = 0.015, compared to the inactive group). We observed similar trends for risk of being overweight (*p*_interaction_ = 0.064 for four levels of physical activity; *p*_interaction_ = 0.043 for the active versus the inactive group).

**Figure 2:**
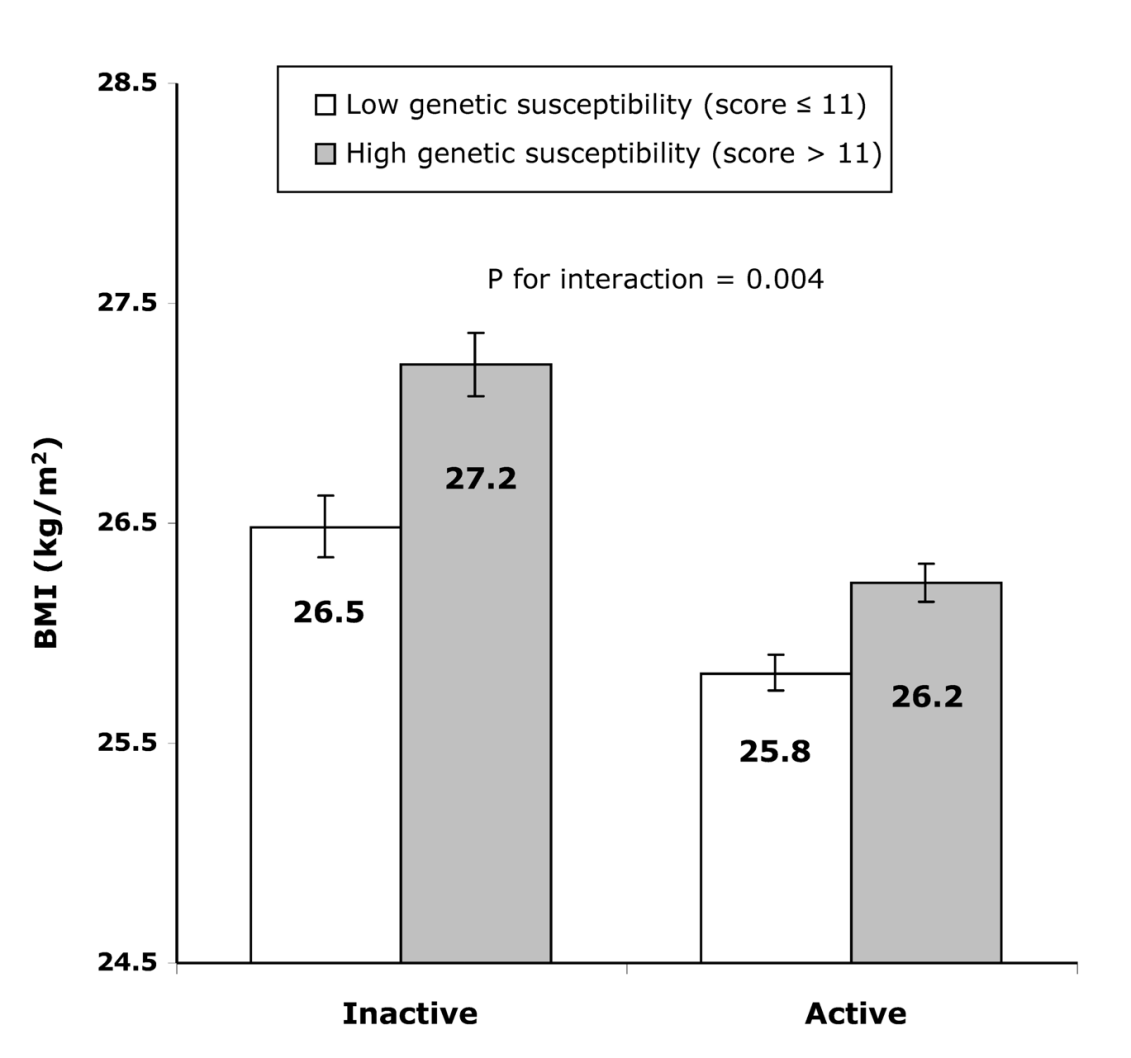
Difference in least square means of BMI between the high (>11 BMI-increasing alleles) and the low (≤11 BMI-increasing alleles) genetic susceptibility groups in the combined active group and the inactive group. Error bars show 95% CIs.

In the inactive group, the difference in BMI between individuals with a high genetic predisposition score (>11 BMI-increasing alleles) and those with a low genetic predisposition score (≤11 BMI-increasing alleles) amounted to 0.739 (SE 0.103) kg/m^2^ (or 2,136 g in weight) (*p*<8.07×10^−13^), whereas this difference was only 0.407 (SE 0.061) kg/m^2^ (or 1,176 g higher weight) (*p*<2.23×10^−11^) in the active group (*p*_interaction_ = 0.004, Figure 2). Similarly, in the inactive group, the odds of obesity were 1.722-fold (95% CI 1.486–1.996; *p* = 2.22×10^−16^) higher in those with a high genetic susceptibility as compared to those with a low genetic susceptibility, while this difference was much smaller (OR 1.287 [95% CI 1.156–1.433; *p* = 1.15×10^−12^]) in the active group (*p*_interaction_ = 0.007).

The genetic predisposition score explained 1.2% of the variation in BMI in the inactive group and 0.6% in the active group. Furthermore, the ROC curves for the prediction of obesity based on the genetic predisposition score together with age, age^2^, sex, showed that the prediction was significantly (*p*<1.00×10^−30^) better in the inactive group (area under the ROC curve, 0.614 [95% CI 0.594–0.635]) than that in the combined active group (0.576 [95% CI 0.561–0.591]).

Of the individual SNP analyses, only rs6548238 near *TMEM18*, rs10838738 in *MTCH2*, and rs7498665 near *SH2B1* showed nominally significant interactions with physical activity level on BMI or obesity risk (Tables S3 and S4), but none survived adjustment for multiple comparisons.

**Figure 3:**
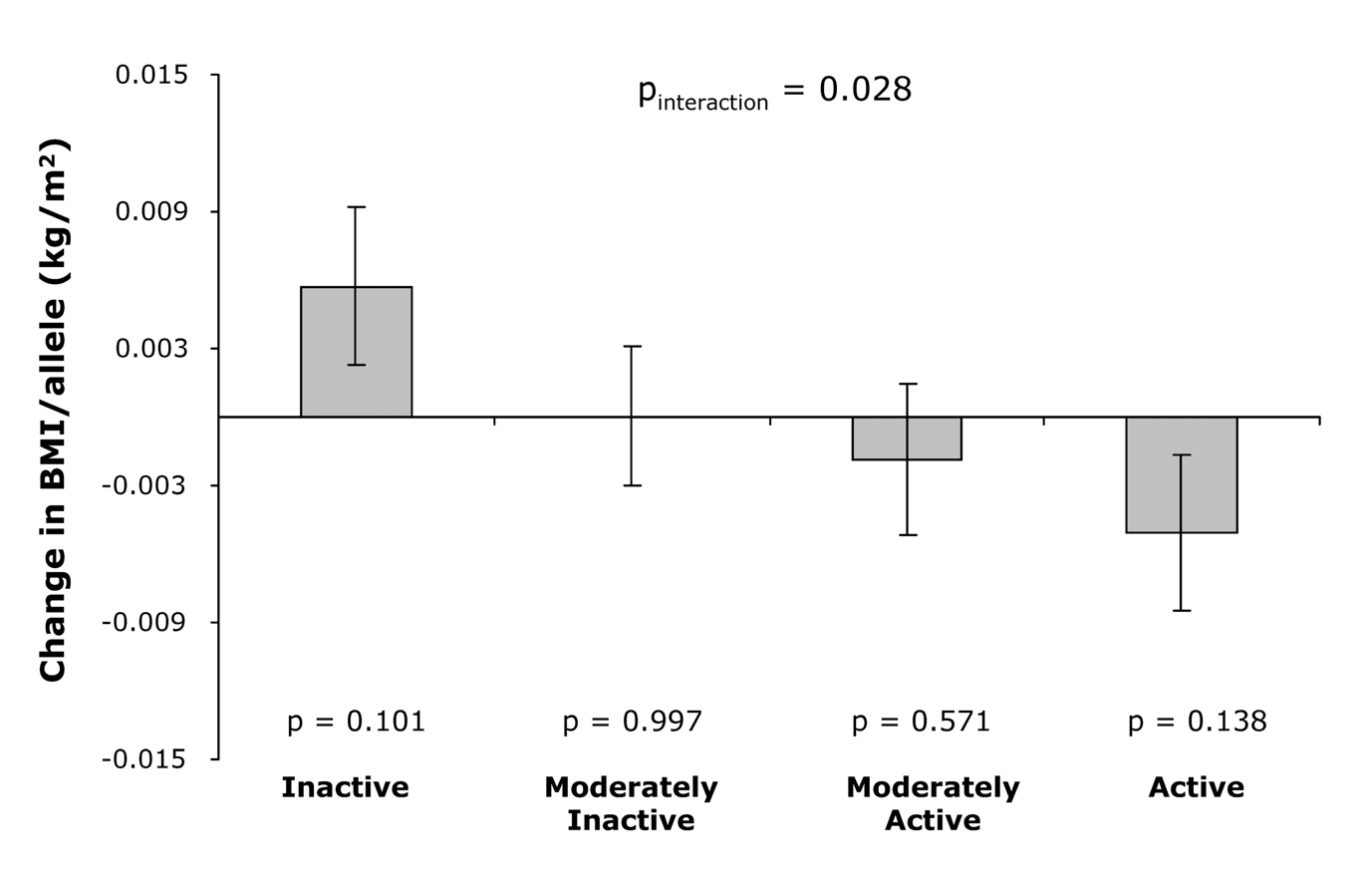
Effect of the genetic predisposition score on the annual change in BMI during follow-up by physical activity level at baseline. Error bars show standard error, and *p*-value at each physical activity level represents the significance of the association between the genetic predisposition score and annual change in BMI.

Consistent with the cross-sectional observations, physical activity modified the association between the genetic predisposition score and annual change in BMI during follow-up (*p*_interaction_ = 0.028, Figure 3). While overall the genetic predisposition score was not associated with the annual BMI change during follow-up (*p* = 0.95), the genetic predisposition score tended to be associated with an increase in annual BMI in physically inactive individuals, whereas the trend was opposite in physically active individuals (*p*_interaction_ = 0.028; Figure 3).

## Discussion

In this analysis of a large-scale population-based study, we show that a physically active lifestyle can modify the genetic predisposition to obesity. On average, each additional obesity-susceptibility allele is associated with an increase in body weight of 445 g. However, in individuals who have a physically active lifestyle, this difference is only 379 g/allele or 36% lower than in physically inactive individuals in whom the difference is 592 g/allele. Consistently, in the total sample each additional obesity-susceptibility allele increases the odds of obesity by 1.116-fold. However, the increased odds per allele for obesity risk are 40% lower in physically active individuals (OR = 1.095) compared to physically inactive individuals (OR = 1.158). We observed the attenuation of the genetic predisposition to obesity already at the lowest levels of physical activity, equivalent to a standing job or a sedentary job with <0.5 h of recreational activity. Importantly, our longitudinal analysis corroborate these cross-sectional observations showing that physical activity significantly (*p*_interaction_ = 0.028) modifies the effect of the genetic predisposition score on the annual BMI change during follow-up. Our findings further emphasise the importance of physical activity in the prevention of obesity.

Preliminary evidence for gene-lifestyle interaction has come from studies on the *FTO* locus, the firstly GWA-identified obesity-susceptibility locus with the largest influence on BMI and obesity risk to date [6],[7],[16]. Several studies have reported that the effect of common *FTO* variants is attenuated in active individuals in different populations [17]–[21]. In some studies, the effect size of *FTO* variants is up to 80% lower in physically active individuals compared to inactive individuals [17],[18],[20]. However, not all studies have been able to demonstrate an *FTO*–physical activity interaction [21]–[25]. This failure to detect an interaction in some studies may reflect the influence of population-specific characteristics such as high overall physical activity levels in the study population [22], small sample size [23],[25], or the effects of age [21]. In our study, the genetic predisposition was estimated by the multiple well-established obesity variants rather than a single locus. While this approach is less informative at a biological level, the greater genetic variation explained by the allele risk score explains why this approach may be preferable in terms of demonstrating an interaction between genetic susceptibility and physical activity.

Our study also showed that variance explained by the genetic predisposition score in the inactive group was 1.2% or twice that observed in the active group (0.6%). This finding is consistent with the increased effect size of the genetic predisposition score on BMI and risk of obesity in the inactive group, and consistent with most of the previous twin studies showing that the genetic contribution to the variation of obesity-related traits, is reduced by increased physical activity levels [26]–[29]. Our finding suggests that gene–environment interactions contribute to the unexplained variance in obesity traits. It also indicates that future GWAS of obesity-related traits may benefit from studying physically inactive individuals because the effect sizes of genetic variants may be more pronounced and therefore easier to identify.

Our data show that increased physical activity levels are associated with lower BMI in the population overall, but that in particular individuals who are genetically predisposed to obesity would benefit more from increased physical activity levels than individuals who are genetically protected. Interventions that target the genetically predisposed may be more effective, a hypothesis to be confirmed in future studies.

The predictive value of the genetic predisposition score for obesity is higher in inactive people, compared to that in the active people. However, even in physically inactive individuals, the extra predictive value provided by the genetic predisposition score beyond information from age and sex is still limited, suggesting that more genetic variants including other forms of variation such as copy number variants and rarer variants remain to be identified. Interactions between these variants and lifestyle factors other than physical activity also need to be examined in future studies.

The strengths of our study include a large sample size, a population-based, prospective study design, and a comprehensive estimation of the genetic predisposition to increased obesity traits based on multiple obesity-susceptibility variants. Previously, we have shown that the identification of convincing gene–environment interactions requires large sample sizes and accurate measurement of genes and environment [30]–[32]. In our study, we combined the strength of a large sample size with a more accurate estimation of the genetic predisposition to obesity. Our results are further strengthened by the longitudinal analysis of BMI change over time. A limitation of our study is that physical activity was measured by a self-administered physical activity questionnaire, which is less accurate than other objective instruments. However, the questionnaire used has been validated and shown to perform well in categorising physical activity levels in this population [15]. Furthermore, we have shown that physical activity assessed by this questionnaire is associated with mortality [33],[34]. Nondifferential measurement error might have attenuated the true strength of the gene–physical activity interaction. We recognise that our longitudinal analysis was limited to a group of individuals who had a lower BMI and were more physically active than the rest of the participants at baseline. However, as the genetic predisposition score was not associated with either physical activity or follow-up status, the selection bias may be limited.

In conclusion, the genetic predisposition to increased BMI and obesity is attenuated by a physically active lifestyle. This attenuation of the genetic predisposition was already observed at low levels of physical activity. Our finding that living a physically active lifestyle is associated with a 40% reduction in the genetic predisposition to common obesity is an important observation for public health. Promoting physical activity, particularly in those who are genetically predisposed, may be an important approach to controlling the current increasing obesity epidemic.

## Supporting Information

Table S1 Comparison of baseline characteristics of participants by follow-up status.
(0.04 MB DOC)

Table S2 Genotype information and quality control statistics for each of the 12 obesity-susceptibility SNPs.
(0.10 MB DOC)

Table S3 Effect size of the 12 SNPs on BMI by physical activity level.
(0.08 MB DOC)

Table S4 OR and 95% CI of the 12 SNPs for obesity by physical activity level.
(0.07 MB DOC)

Alternative Language Abstract S1 Translation of the Abstract into Chinese-Mandarin by Shengxu Li.
(0.03 MB DOC)

Alternative Language Abstract S2 Translation of the Abstract into Dutch by Ruth Loos.
(0.04 MB DOC)

## Abbreviations

BMI: body mass index
CI: confidence interval
EPIC: European Prospective Investigation of Cancer
GWAS: genome-wide association studies
OR: odds ratio
ROC: receiver operating characteristic
SE: standard error
