# Supplementary material for: Physical Activity Attenuates the Genetic Predisposition to Obesity in 20,000 Men and Women from EPIC-Norfolk Prospective Population Study
Source: PLoS Med. 2010 Aug 31;7(8):e1000332. doi: 10.1371/journal.pmed.1000332 (PMC2930873; doi:10.1371/journal.pmed.1000332)
Supplement: Table S1 — Comparison of baseline characteristics of participants by follow-up status. (0.04 MB DOC) [file pmed.1000332.s001.doc]

**Table S1.** Comparison of baseline characteristics of participants by follow-up status

|  | Follow-up | | p |
| --- | --- | --- | --- |
|  | Yes | No |  |
| **N** | 11,936 | 6,004 |  |
| Age (years)* | 58.7 ± 8.9 | 58.8 ± 9.8 | 0.212 |
| BMI (kg/m^2^)* | 26.0 ± 3.5 | 26.6 ± 4.0 | 1.06×10^-33^ |
| Men/Women (n, %) | 5,969/5,967 (50.0/50.0) | 4,035/4,459 (47.5/52.5) | 0.0004 |
| Physical activity level | N (%) | N (%) |  |
| Inactive | 3202 (26.8) | 2964 (34.9) | 3.85×10^-36^ |
| Moderately inactive | 3458 (29.0) | 2369 (27.9) |  |
| Moderately active | 2909 (24.4) | 1747 (20.6) |  |
| Active | 2367 (19.8) | 1414 (16.6) |  |

*: Values represent mean ± SD.
