# Supplementary material for: Physical Activity Attenuates the Genetic Predisposition to Obesity in 20,000 Men and Women from EPIC-Norfolk Prospective Population Study
Source: PLoS Med. 2010 Aug 31;7(8):e1000332. doi: 10.1371/journal.pmed.1000332 (PMC2930873; doi:10.1371/journal.pmed.1000332)
Supplement: Table S2 — Genotype information and quality control statistics for each of the 12 obesity-susceptibility SNPs. (0.10 MB DOC) [file pmed.1000332.s002.doc]

**Table S2.** Genotype information and quality control statistics for each of the 12 obesity-susceptibility SNPs

| **SNP** | **Nearest gene** | **Chromosome** | **Position (bp)*** | **Risk allele** | **Non-risk allele** | **Risk allele frequency**  **(%)** | **HWE test**  **p-value** | **Original reported leading SNP** | **References** | **r^2^ between the genotyped and original SNP** |
| --- | --- | --- | --- | --- | --- | --- | --- | --- | --- | --- |
| rs3101336 | *NEGR1* | 1 | 72,523,206 | C | T | 0.61 | 0.27 | rs2815752 | [8] | 1.00 |
| rs10913469 | *SEC16B* | 1 | 176,180,142 | G | A | 0.20 | 0.63 | rs10913469 | [9] |  |
| rs6548238 | *TMEM18* | 2 | 624,905 | C | T | 0.83 | 0.88 | rs6548238 | [8] |  |
| rs7647305 | *ETV5* | 3 | 187,316,992 | C | T | 0.79 | 0.08 | rs7647305 | [9] |  |
| rs10938397 | *GNPDA2* | 4 | 45,023,455 | G | A | 0.42 | 0.14 | rs10938397 | [8] |  |
| rs925946 | *BDNF* | 11 | 27,623,778 | T | G | 0.31 | 0.73 | rs925946 | [9] |  |
| rs10838738 | *MTCH2* | 11 | 47,619,625 | G | A | 0.34 | 0.15 | rs10838738 | [8] |  |
| rs7132908 | *FAIM2* | 12 | 48,549,415 | T | C | 0.39 | 0.37 | rs7138803 | [9] | 0.94 |
| rs7498665 | *SH2B1* | 16 | 28,790,742 | G | A | 0.40 | 0.69 | rs7498665 | [8] |  |
| rs1121980 | *FTO* | 16 | 52,366,748 | A | G | 0.43 | 0.72 | rs9939609 | [5] | 0.84 |
| rs17782313 | *MC4R* | 18 | 56,002,077 | C | T | 0.23 | 0.38 | rs17782313 | [7] |  |
| rs368794 | *KCTD15* | 19 | 3,9012,292 | A | T | 0.67 | 0.11 | rs11084753 | [8] | 1.00 |

HWE: Hardy-Weinberg Equilibrium

*: Build 35 position

Maximum n = 20,125 for individual SNPs
