# Supplementary material for: Physical Activity Attenuates the Genetic Predisposition to Obesity in 20,000 Men and Women from EPIC-Norfolk Prospective Population Study
Source: PLoS Med. 2010 Aug 31;7(8):e1000332. doi: 10.1371/journal.pmed.1000332 (PMC2930873; doi:10.1371/journal.pmed.1000332)
Supplement: Table S3 — Effect size of the 12 SNPs on BMI by physical activity level. (0.08 MB DOC) [file pmed.1000332.s003.doc]

**Table S3.** Effect size of the 12 SNPs on BMI by physical activity level

| SNP | Nearest gene | Inactive | | |  | Moderately Inactive | | |  | Moderately Active | | |  | Active | | |  | p for interaction |
| --- | --- | --- | --- | --- | --- | --- | --- | --- | --- | --- | --- | --- | --- | --- | --- | --- | --- | --- |
|  |  | beta | se | p |  | beta | se | p |  | beta | se | p |  | beta | se | p |  |  |
| rs3101336 | *NEGR1* | 0.144 | 0.075 | 0.054 |  | 0.071 | 0.071 | 0.317 |  | 0.002 | 0.078 | 0.984 |  | 0.099 | 0.082 | 0.225 |  | 0.446 |
| rs10913469 | *SEC16B* | 0.191 | 0.094 | 0.042 |  | 0.140 | 0.087 | 0.107 |  | 0.067 | 0.097 | 0.493 |  | 0.193 | 0.101 | 0.056 |  | 0.837 |
| rs6548238 | *TMEM18* | 0.456 | 0.097 | 2.82×10^-6^ |  | 0.142 | 0.094 | 0.130 |  | 0.246 | 0.099 | 0.014 |  | 0.081 | 0.106 | 0.443 |  | 0.019 |
| rs7647305 | *ETV5* | 0.026 | 0.089 | 0.771 |  | -0.006 | 0.086 | 0.942 |  | 0.072 | 0.096 | 0.451 |  | 0.246 | 0.099 | 0.013 |  | 0.096 |
| rs10938397 | *GNPDA2* | 0.169 | 0.076 | 0.026 |  | 0.185 | 0.073 | 0.011 |  | 0.169 | 0.080 | 0.035 |  | 0.121 | 0.083 | 0.145 |  | 0.654 |
| rs925946 | *BDNF* | 0.232 | 0.082 | 0.005 |  | 0.251 | 0.080 | 0.002 |  | 0.111 | 0.085 | 0.191 |  | 0.252 | 0.089 | 0.005 |  | 0.701 |
| rs10838738 | *MTCH2* | 0.188 | 0.079 | 0.017 |  | 0.021 | 0.075 | 0.782 |  | 0.168 | 0.083 | 0.043 |  | -0.135 | 0.087 | 0.122 |  | 0.040 |
| rs7132908 | *FAIM2* | 0.164 | 0.077 | 0.032 |  | 0.139 | 0.072 | 0.052 |  | 0.110 | 0.079 | 0.165 |  | 0.219 | 0.083 | 0.008 |  | 0.804 |
| rs7498665 | *SH2B1* | 0.194 | 0.074 | 0.009 |  | 0.061 | 0.070 | 0.385 |  | 0.074 | 0.077 | 0.333 |  | -0.010 | 0.082 | 0.900 |  | 0.076 |
| rs1121980 | *FTO* | 0.460 | 0.075 | 9.55×10^-10^ |  | 0.246 | 0.071 | 0.001 |  | 0.285 | 0.079 | 3.11×10^-4^ |  | 0.281 | 0.080 | 4.80×10^-4^ |  | 0.113 |
| rs17782313 | *MC4R* | 0.136 | 0.087 | 0.120 |  | 0.223 | 0.082 | 0.007 |  | 0.211 | 0.090 | 0.019 |  | 0.164 | 0.095 | 0.084 |  | 0.836 |
| rs368794 | *KCTD15* | 0.049 | 0.080 | 0.539 |  | 0.180 | 0.074 | 0.015 |  | 0.049 | 0.082 | 0.549 |  | -0.002 | 0.085 | 0.985 |  | 0.480 |

p values were adjusted for age, age^2^, and sex.

p for interaction: test the interaction between each SNP and physical activity levels (4 groups) on BMI.
