# Supplementary material for: Physical Activity Attenuates the Genetic Predisposition to Obesity in 20,000 Men and Women from EPIC-Norfolk Prospective Population Study
Source: PLoS Med. 2010 Aug 31;7(8):e1000332. doi: 10.1371/journal.pmed.1000332 (PMC2930873; doi:10.1371/journal.pmed.1000332)
Supplement: Table S4 — OR and 95% CI of the 12 SNPs for obesity by physical activity level. (0.07 MB DOC) [file pmed.1000332.s004.doc]

**Table S4.** Odds ratio and 95% confidence interval of the 12 SNPs for obesity by physical activity level

| SNP | Nearest gene | Inactive | | |  | Moderately Inactive | | |  | Moderately Active | | |  | Active | | |  | p for interaction |
| --- | --- | --- | --- | --- | --- | --- | --- | --- | --- | --- | --- | --- | --- | --- | --- | --- | --- | --- |
|  |  | OR | 95% lower limit | 95% upper limit |  | OR | 95% lower limit | 95% upper limit |  | OR | 95% lower limit | 95% upper limit |  | OR | 95% lower limit | 95% upper limit |  |  |
| rs3101336 | *NEGR1* | 1.107 | 0.994 | 1.233 |  | 1.015 | 0.900 | 1.144 |  | 0.954 | 0.832 | 1.095 |  | 1.043 | 0.889 | 1.2225 |  | 0.226 |
| rs10913469 | *SEC16B* | 1.085 | 0.950 | 1.239 |  | 0.994 | 0.853 | 1.158 |  | 0.972 | 0.820 | 1.153 |  | 1.087 | 0.895 | 1.319 |  | 0.755 |
| rs6548238 | *TMEM18* | 1.295 | 1.123 | 1.493 |  | 1.136 | 0.965 | 1.339 |  | 1.287 | 1.071 | 1.548 |  | 1.022 | 0.834 | 1.253 |  | 0.176 |
| rs7647305 | *ETV5* | 1.063 | 0.935 | 1.209 |  | 1.004 | 0.866 | 1.164 |  | 1.099 | 0.926 | 1.304 |  | 1.306 | 1.067 | 1.598 |  | 0.094 |
| rs10938397 | *GNPDA2* | 1.154 | 1.035 | 1.286 |  | 1.158 | 1.021 | 1.313 |  | 1.115 | 0.967 | 1.286 |  | 1.142 | 0.974 | 1.340 |  | 0.801 |
| rs925946 | *BDNF* | 1.165 | 1.037 | 1.308 |  | 1.201 | 1.050 | 1.374 |  | 1.097 | 0.944 | 1.276 |  | 1.199 | 1.011 | 1.421 |  | 0.975 |
| rs10838738 | *MTCH2* | 1.116 | 0.999 | 1.247 |  | 1.000 | 0.878 | 1.139 |  | 1.049 | 0.906 | 1.215 |  | 0.906 | 0.765 | 1.073 |  | 0.084 |
| rs7132908 | *FAIM2* | 1.153 | 1.034 | 1.285 |  | 1.106 | 0.978 | 1.251 |  | 1.047 | 0.912 | 1.203 |  | 1.099 | 0.940 | 1.285 |  | 0.405 |
| rs7498665 | *SH2B1* | 1.218 | 1.094 | 1.356 |  | 1.047 | 0.929 | 1.181 |  | 1.100 | 0.962 | 1.259 |  | 0.975 | 0.833 | 1.141 |  | 0.029 |
| rs1121980 | *FTO* | 1.399 | 1.255 | 1.560 |  | 1.182 | 1.046 | 1.335 |  | 1.297 | 1.127 | 1.498 |  | 1.280 | 1.096 | 1.495 |  | 0.434 |
| rs17782313 | *MC4R* | 1.131 | 1.001 | 1.279 |  | 1.184 | 1.030 | 1.362 |  | 1.162 | 0.995 | 1.358 |  | 1.132 | 0.947 | 1.353 |  | 0.896 |
| rs368794 | *KCTD15* | 0.964 | 0.861 | 1.079 |  | 1.197 | 1.051 | 1.363 |  | 1.008 | 0.873 | 1.163 |  | 1.018 | 0.865 | 1.197 |  | 0.793 |

p values were adjusted for age, age^2^, and sex.

p for interaction: test the interaction between each SNP and physical activity levels (4 groups) on risk of obesity.
