# Supplementary material for: Physical Activity Attenuates the Genetic Predisposition to Obesity in 20,000 Men and Women from EPIC-Norfolk Prospective Population Study
Source: PLoS Med. 2010 Aug 31;7(8):e1000332. doi: 10.1371/journal.pmed.1000332 (PMC2930873; doi:10.1371/journal.pmed.1000332)
Supplement: Alternative Language Abstract S1 — Translation of the Abstract into Chinese-Mandarin by Shengxu Li. (0.03 MB DOC) [file pmed.1000332.s005.doc]

**体力活动降低肥胖遗传易感性的影响：英国EPIC-Norfolk前瞻性研究**

李升绪 赵京华 栾建安 沃夫 · 埃克伦德 罗伯特 · 鲁宾
许珂琪 尼科拉斯 · 韦勒姆 露丝 · 洛斯

英国医学研究院流行病学研究所，剑桥代谢科学研究所

剑桥大学公共卫生研究所公共卫生和初级保健系

**背景：**我们先前的研究表明，由全基因组关联研究(GWA)所发现的多个遗传位点以累积性的方式增加对普通肥胖的遗传易感性。但是，这种遗传易感性的影响是否会被活跃的体力活动所减低并不清楚。在本研究中，我们评价了体力活动和肥胖遗传易感性对肥胖相关指标的影响之间的关系。

**方法与结果：**我们对欧洲前瞻性癌症研究中来自英国诺福克地区(Norfolk)的20,430人进行了随访(平均随访时间为3.6年)。对每个被随访者，我们对其12个单核苷酸多态性(SNP，均由全基因组关联研究发现) 标记进行了基因分型并根据这些遗传标记计算其遗传易感性积分。我们采用调查表评价被随访者的个人体力活动是否活跃。统计分析中，我们用线性和logistic回归模型来评价遗传易感性积分的主效应以及与体力活动的交互作用对体重指数相关指标的影响。在此模型中，12个SNP的作用均设定为叠加性。经分析发现，在观察人群中，每增加一个遗传易感性积分，体重指数平均增加0.154个单位（对一个1.7米高的人，相当于0.45 公斤体重）。然而在体力活动不活跃的人当中，这一增加量为0.205个单位（相当于0.59公斤体重），在活跃的人当中，这个增加量仅为0.131个单位 （相当于0.38公斤体重）(p_交互作用_=0.005)。类似地，每增加一个遗传易感性积分，肥胖危险增加11.6%，但增加的幅度在体力活动不活跃的人中达到15.8%，在活跃的人中只有9.5%。体力活动对体重指数变化与遗传易感性积分之间的关联起修饰作用(p_交互作用_=0.028)，这与横断面调查结果一致，。

**结论：**我们的研究表明活跃的体力活动会降低普通肥胖遗传易感性的影响。

**关键词：**体重指数，肥胖，遗传易感性，体力活动，交互作用
