# Supplementary material for: Physical Activity Attenuates the Genetic Predisposition to Obesity in 20,000 Men and Women from EPIC-Norfolk Prospective Population Study
Source: PLoS Med. 2010 Aug 31;7(8):e1000332. doi: 10.1371/journal.pmed.1000332 (PMC2930873; doi:10.1371/journal.pmed.1000332)
Supplement: Alternative Language Abstract S2 — Translation of the Abstract into Dutch by Ruth Loos. (0.04 MB DOC) [file pmed.1000332.s006.doc]

**Fysieke activiteit vermindert de genetische voorbeschiktheid tot obesiteit in 20,000 mannen en vrouwen van de EPIC-Norfolk studie**

Shengxu Li^1^, Jing Hua Zhao^1^, Jian’an Luan^1^, Ulf Ekelund^1^, Robert N Luben^2^, Kay-Tee Khaw^2^, Nicholas J Wareham^1^, Ruth J.F Loos^1^

1 MRC Epidemiology Unit, Institute of Metabolic Science, Cambridge CB2 0QQ, UK

2 Department of Public Health and Primary Care, Institute of Public Health, University of Cambridge, Cambridge CB2 0SR, UK

***Achtergrond:*** Wij toonden eerder al aan dat meerdere genetische varianten, die geindentificeerd werden met genoom-wijde associatie studies, de voorbeschiktheid tot obesiteit op een cumulatieve wijze vergroot. Het is echter niet geweten of deze genetische voorbeschiktheid vermindert wanneer men een fysiek actieve levensstijl leidt. Daarom bestudeerden we in deze studie de invloed van een fysiek actieve levensstijl op de genetische voorbeschiktheid tot obesiteit in een grote populatie uit Groot Britannië.

***Methodes:*** We bepaalden de genotypes (allelen) van 12 genetische varianten, die eerder werden geindentificeerd met genoom-wijde analyses voor BMI, in een populatie van 20,430 individuen (leeftijd 39-79 jaar) van de European Prospective Investigation of Cancer (EPIC)-Norfolk studie. Een genetische voorbeschiktheids-score werd berekent door, voor elk individu, het aantal BMI-verhoogende allelen van deze 12 varianten op te tellen. Fysieke activiteit werd bepaald aan de hand van een vragenlijst. Lineaire and logistische regressie modellen werden gebruikt om de invloed van de genetische voorbeschiktheids-score en de interacties met fysieke activiteit op BMI and obesiteit te bepalen.

***Bevindingen*** Elk bijkomend allel was geassocieerd met een 0.154 kg/m^2^ (p=6.73×10^-37^) hogere BMI (gelijkwaardig met 445g in lichaamsgewicht voor een persoon van 1.70m groot). Deze relatie was significant (p_interaction_=0.005) meer uitgesproken in inactieve personen [0.205 kg/m^2^ (p=3.62×10^-18^; 592g in lichaamsgewicht)] dan in actieve personen [0.131 kg/m^2^ (p=7.97×10^-21^; 379g in lichaamsgewicht)]. Verder was elk bijkomend allel ook geassocieerd met een 11.6% (p=3.37×10^-26^) hoger risico op obesiteit in de totale populatie, maar significant (p_interaction_=0.015) meer in inactieve personen [15.8%; p=1.93×10^-16^)] dan in actieve personen [9.5%; p=1.15×10^-12^)].

***Conclusies:*** Onze study toont aan dat een fysiek actieve levensstijl geassocieerd is met een 40% vermindering van de genetische voorbeschiktheid tot obesiteit.
